# A Systematic Review and Meta-analysis of School-Based Preventive Interventions Targeting E-Cigarette Use Among Adolescents

**DOI:** 10.1007/s11121-024-01730-6

**Published:** 2024-09-26

**Authors:** Lauren A. Gardner, Amy-Leigh Rowe, Nicola C. Newton, Lyra Egan, Emily Hunter, Emma K. Devine, Tess Aitken, Louise Thornton, Maree Teesson, Emily Stockings, Katrina E. Champion

**Affiliations:** 1 The Matilda Centre for Research in Mental Health and Substance Use, University of Sydney, Sydney, Australia; 2 University of Sydney Library, University of Sydney, Sydney, Australia; 3 School of Medicine and Public Health, The University of Newcastle, Newcastle, Australia

**Keywords:** E-cigarettes, Vaping, School, Intervention, Prevention

## Abstract

This study aimed to examine the efficacy of school-based e-cigarette preventive interventions via a systematic review and meta-analysis. We searched Medline, Embase, PsycINFO, Scopus, CINAHL, Cochrane, and clinical trial registries for studies published between January 2000 and June 2023 using keywords for e-cigarettes, adolescents, and school. Of 1566 double-screened records, 11 met the criteria of targeting adolescents, evaluating an e-cigarette preventive intervention, being conducted in a secondary school, using a randomized controlled trial (RCT), cluster RCT, or quasi-experimental design, and comparing an intervention to a control. Pre-specified data pertaining to the study design, outcomes, and quality were extracted by one reviewer and confirmed by a second, and where necessary, a third reviewer. Meta-analyses found no evidence that school-based interventions prevented e-cigarette use at the longest follow-up, which ranged between 6 and 36 months post-intervention (OR = 0.43, 95% CI = 0.16, 1.12; *p* = 0.09). However, subgroup analyses identified significant effects at post-test and when studies with < 12-month follow-up were omitted. No effect was found for tobacco use at the longest follow-up (OR = 1.01, 95% CI = 0.65, 1.59, *p* = 0.95); however, reductions in past 30-day tobacco use (OR = 0.59, 95% CI = 0.39, 0.89, *p* = 0.01) which encompassed e-cigarettes in some studies were identified. Narrative synthesis supported these mixed results and found some school-based interventions prevented or reduced e-cigarette and/or tobacco use; however, some increased use. School-based interventions were also associated with improved knowledge (SMD =  − 0.38, 95% CI =  − 0.68, − 0.08, *p* = 0.01), intentions (SMD =  − 0.15, 95% CI =  − 0.22, − 0.07, *p* = 0.0001), and attitudes (SMD =  − 0.14, 95% CI =  − 0.22, − 0.06; *p* = 0.0007) in the short term. Overall, the quality of evidence was low-to-moderate. School-based interventions hold the potential for addressing e-cigarette use, however, can have null or iatrogenic effects. More high-quality research is needed to develop efficacious interventions, and schools must be supported to adopt evidence-based programs**.** This is the first systematic review and meta-analysis to examine the efficacy of school-based preventive interventions for e-cigarette use. It provides crucial new knowledge about the efficacy of such interventions in preventing e-cigarette use and improving other outcomes (e.g., tobacco use, knowledge, intentions, attitudes, and mental health) among adolescents and the key characteristics associated with efficacious interventions. Our findings have important practical implications, highlighting future research directions for the development and evaluation of e-cigarette preventive interventions, along with the need to provide support to schools to help them identify and adopt evidence-based programs.

**Supplementary Information:**

The online version contains supplementary material available at 10.1007/s11121-024-01730-6.

The growing use of electronic cigarettes (“e-cigarettes,” also known as “vapes”) among adolescents (i.e., those aged 10–19 years) is a critical public health concern due to the considerable potential for harm. For example, e-cigarettes can cause a range of health issues including respiratory disease, seizures, poisoning, and injuries (Banks et al., 2023). E-cigarettes have also been associated with adverse mental health outcomes among adolescents including depression and suicidal ideation, with relatively little known about the longer-term health effects (Banks et al., 2023; Gardner et al., 2024; Truong & Cotton, 2023). Additionally, adolescents are particularly susceptible to nicotine addiction, putting the developing brain at risk of damage (Banks et al., 2023; Yuan et al., 2015). In Australia, approximately one in four adolescents aged 14–17 have tried e-cigarettes, with one in ten reporting past 30-day use (Gardner et al., 2023). Rates of past 30-day use are also high among adolescents in Canada and the United States, at 13% and 10%, respectively (Birdsey et al., 2023; Statistics Canada, 2023). Among 11–18-year-olds in the UK, 40% have used e-cigarettes, and approximately 5% use at least monthly (Vrinten et al., 2023). Evidence suggests that e-cigarette use is also associated with an increased likelihood of tobacco cigarette smoking (Soneji et al., 2017; Yoong et al., 2021). This is highly concerning, given the substantial health, economic, and societal burden of tobacco smoking (Rethink Addiction & KPMG, 2022; Yoong et al., 2021). Effective preventive interventions and public health strategies are therefore urgently needed to address e-cigarette use among adolescents.

While policy-level prevention initiatives can be effective and continue to evolve, they are unlikely to eliminate e-cigarette use completely. For example, there is a minimum age of purchase regulations in the United States (US), with some US states aiming to reduce access, supply, and use via banning online sales and flavors (US Food & Drug Administration, 2020). Australia has adopted a strict therapeutic-only model, whereby sales of nicotine-containing e-cigarettes are restricted to those aged over 18 via community pharmacies,^1^ with the intention that they are used to quit smoking (Parliament of Australia, 2024). Despite the varied measures, rates of e-cigarette use among adolescents in these regions remain high, highlighting the need to provide critical education and resistance skills training to prevent or reduce the harms from e-cigarette use (Stockings et al., 2024).

School-based interventions are an efficient, effective, and economical prevention strategy. Schools offer an opportune setting to reach large numbers of adolescents during a critical period of exposure to substance use and in an environment designed for learning and shaping of behavior (Guerin & White, 2018; Tully, 2007). Further, adolescents’ social lives often revolve around the school context, meaning education can be delivered to peer groups whom can be strong influences on substance use behaviors (Guerin & White, 2018; Tully, 2007). Substance use preventive interventions are also feasible to implement in the school context, given drug education is typically mandatory within health education curriculums (Australian Curriculum Assessment and Reporting Authority, 2022). Evidence shows that school-based interventions can be effective at preventing and/or delaying the use of various substances among adolescents, including tobacco, alcohol, and other drugs (Thomas et al., 2015; Tully, 2007; Wiehe et al., 2005), with some interventions demonstrating sustained effects into early adulthood (Newton et al., 2022). As adolescent e-cigarette use has grown rapidly in recent years, so too have the number of interventions aiming to prevent such use. However, to our knowledge, no study has systematically examined and meta-analyzed the efficacy of school-based preventive interventions for e-cigarette use. Additionally, there is a need to understand the key characteristics of efficacious interventions, such as the program type (e.g., skills-based classroom programs vs parent information vs school community programs), mode of delivery (e.g., eHealth vs face-to-face), intervention duration and frequency, portion dedicated to e-cigarettes (e.g., specific e-cigarette programs versus broader substance use), and theoretical underpinnings to guide future intervention development and refinement, and to advise program delivery in school settings. This study therefore aims to.

1. Evaluate the efficacy of school-based preventive interventions in preventing e-cigarette use and improving secondary outcomes (e.g., e-cigarette-related knowledge, intentions, and attitudes, tobacco use, and mental health) among adolescents.
2. Identify and summarize the key components and characteristics associated with efficacious interventions.

## Methods

### Approach and Search Terminology

This systematic review was conducted in accordance with the published review protocol (Gardner et al., 2022), the Preferred Reporting Items for Systematic Reviews and Meta-Analyses (PRISMA) guidelines (Moher et al., 2015) and the Synthesis Without Meta-Analysis Guidelines (SWiM) (Campbell et al., 2020), and was prospectively registered with the International Prospective Register of Systematic Reviews (PROSPERO; CRD42022323352).

A research librarian (TA) conducted a systematic search of Medline (Ovid), Embase (Ovid), PsycINFO (Ovid), Scopus, CINAHL, the Cochrane Database of Systematic Reviews (Ovid), and international clinical trial registries via the Cochrane Central Register of Controlled Trials (Ovid) for studies published from January 2000 (to slightly precede the advent of e-cigarettes in 2003 and thus ensure full capture of studies) to June 2022, which was re-run in June 2023. The search was limited to human studies, had no language restrictions, and was designed to identify gray literature and unpublished work, including dissertations, clinical trial registries, and conference proceedings. An example search strategy is provided in Supplemental Table S1. All records identified in the search strategy were exported into EndNote and uploaded to the Covidence online software program for deduplication and screening. Reference lists of eligible papers were reviewed to identify other relevant studies.

#### Eligibility Criteria

To be eligible for inclusion, studies must have (1) targeted adolescents aged between 11 and 18 years of age at study intake (i.e., those of secondary school age); (2) evaluated an intervention targeting the prevention of e-cigarette use; (3) been conducted in a secondary school setting, however, school-based interventions incorporating additional components (such as family-based or community-based elements) were also eligible; (4) used a randomized controlled trial, cluster randomized controlled trial, or a quasi-experimental design; and (5) compared an intervention group to a control group who did not receive an intervention, education as usual, or alternative intervention. Interventions addressing other risk behaviors in addition to e-cigarette use (e.g., tobacco use or illicit drug use) and of both universal (i.e., delivered to all students in the intervention condition, regardless of their level of risk) and selective (i.e., delivered to higher-risk students in the intervention condition) nature were eligible.

#### Exclusion Criteria

Studies were excluded on the basis of (1) not targeting adolescents, (2) not directly addressing e-cigarette use in the intervention, (3) the intervention having no school-based components, and (4) having a non-experimental design or no control group.

### Article Screening and Coding

**Fig. 1:**
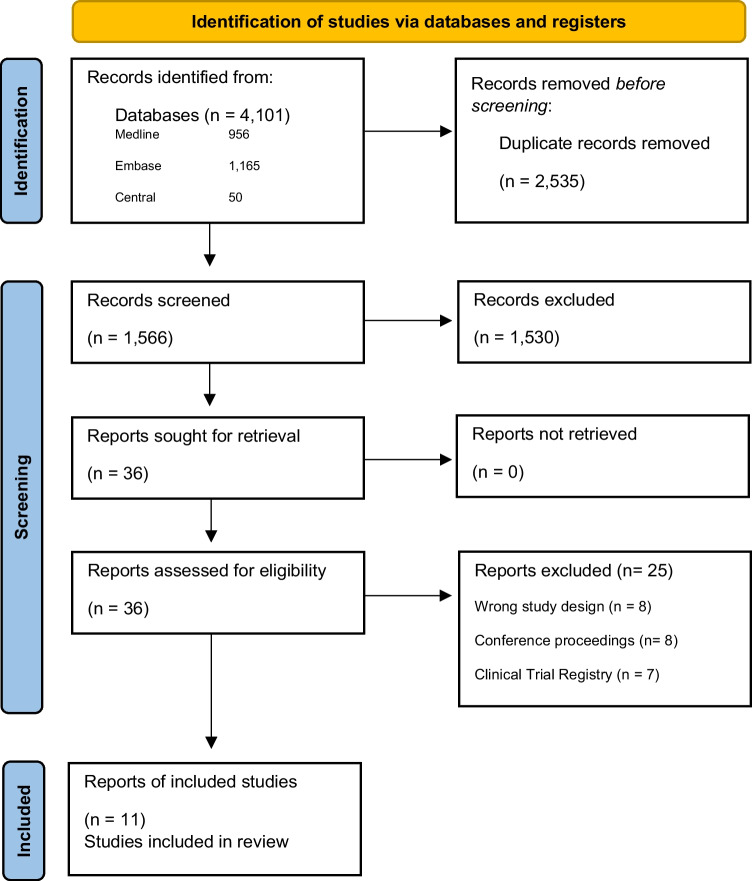
PRISMA flow diagram

Two reviewers (LAG and AR) independently screened 1566 title and abstract records (98% agreement), as well as 36 full-text records (94% agreement). Discrepancies at both screening stages were resolved by a third reviewer (KEC), resulting in 11 articles eligible for inclusion (see Fig. 1 PRISMA flow diagram). Data were extracted by one reviewer (EH or LE) and confirmed by a second (LAG), and where necessary, a third (ES) reviewer. The following information from each eligible article was extracted: publication details, study characteristics, participant characteristics, intervention characteristics (including program type, intervention duration/frequency, delivery method [e.g., face-to-face, online, hybrid], portion dedicated to e-cigarettes, and theoretical underpinnings), primary and secondary outcomes of interest across all timepoints, measurement tools employed, details of the comparison group, data to assess risk of bias, and process data to determine the degree to which an intervention was implemented as intended, including adherence/attendance, where reported. Corresponding authors were contacted for missing data. Further information on data extraction can be found in the published protocol (Gardner et al., 2022).

### Data Analysis

#### Primary and Secondary Outcomes

The primary outcome was the prevention of e-cigarette use (lifetime use/ever use) at the longest follow-up among adolescents reporting never using e-cigarettes at baseline. Secondary outcomes included the reduction or cessation of e-cigarette use (past 30-day) among adolescents already reporting e-cigarette use at baseline; the prevention (lifetime use/ever use) and reduction/cessation (past 30-day) of tobacco cigarette use; knowledge, attitudes (termed “outcome expectations” in some studies), normative beliefs, harm perceptions, and future intentions related to e-cigarette/tobacco use; mental health outcomes; and other substance use.

#### Variable Definition and Data Transformation

Prevention of e-cigarette use at the longest follow-up (primary outcome), reduction or cessation of e-cigarette use (past 30-day), and the prevention (lifetime use/ever use) and reduction/cessation (past 30-day) of tobacco cigarette use were treated as dichotomous variables where *N* of participants in the intervention group reporting e-cigarette/cigarette use was compared with *N* of participants in the control group reporting e-cigarette/cigarette use. Raw *N*s were extracted or reverse engineered, where possible; otherwise, effect sizes and their confidence intervals were extracted. All other outcomes (knowledge, attitudes, normative beliefs, harm perceptions, future intentions, mental health, and other substance use) were treated as continuous outcomes expressed as means and standard deviations. Where standard deviations were not reported, they were calculated from other data reported in the study. To correct for inconsistent directionality of continuous outcomes (e.g., where higher scores indicate higher knowledge in one study but lower knowledge in another), scores were inverted (multiplied by − 1) where required to ensure all scales pointed in the same direction prior to meta-analysis (Higgins et al., 2023). This occurred for two secondary outcomes: knowledge and harm perceptions. If a study reported data at multiple timepoints, e-cigarette/tobacco use outcomes were meta-analyzed at the longest follow-up timepoint, as per our pre-specified primary outcome and given substance use behavior change is more likely to be observed over the longer time period, as exposure increases throughout adolescence (Guerin & White, 2018; Thomas et al., 2015). Conversely, outcomes theorized to precede behavior change and exhibit change over the shorter term (e.g., knowledge, attitudes, beliefs, and intentions) were examined at the post-test timepoint (Ajzen, 1991; Bandura, 1986).

#### Meta-analysis of Quantitative Data

To address Aim 1—evaluate the efficacy of school-based preventive interventions in preventing e-cigarette use and improving secondary outcomes—data were meta-analyzed via inverse variance weighted random-effects analysis in RevMan (version 6.7.1, Cochrane, London, UK). Dichotomous outcomes were expressed as odds ratios (ORs) and continuous outcomes as standardized mean differences (SMDs), with associated 95% confidence intervals (CIs). For dichotomous outcomes, ORs < 1 indicate lower odds of the outcome in the intervention group relative to control, and ORs > 1 favor control. Results are presented using forest plots, and heterogeneity was assessed using the Higgins *I*^2^ statistic (with values of 25%, 50%, and 75%, representing small, moderate, or large heterogeneity, respectively). Tau^2^ and prediction intervals were calculated to assess between-study variance in the underlying effect estimates (Campbell et al., 2020). The significance of any heterogeneity was examined using Cochran’s *Q* (*χ*^2^) test (*p* < 0.05). Where appropriate, sub-group analyses were conducted to explore sources of significant heterogeneity based on study factors, including follow-up timepoint, intervention type, risk of bias, and study design.

#### Narrative Synthesis of Qualitative Data

To address Aim 2—summarize the key components and characteristics associated with efficacious interventions—a narrative synthesis of study and intervention characteristics was conducted in line with the SWiM guidelines (Campbell et al., 2020), as per our protocol (Gardner et al., 2022). Efficacious interventions were defined as those with programs demonstrating a significant difference (at *p* < 0.05) between the intervention and control groups on at least one primary or secondary outcome. As per SWiM guidelines and dependent on data availability, we synthesized the following characteristics of efficacious interventions for each primary and secondary outcome: delivery method (e.g., eHealth vs face-to-face, peer- vs teacher-led); intervention duration, frequency, and adherence; theoretical underpinning of intervention; program type (e.g., classroom education vs broader community initiative); and portion of program dedicated to e-cigarette use (e.g., entire program vs supplemental section).

#### Risk of Bias and Quality of Evidence

Two authors (AR and EKD [first search]; AR and LAG [updated search]) independently conducted the risk of bias assessments for all studies. Randomized studies were assessed using the Cochrane Collaboration’s tool for assessing risk of bias (RoB 2) (Higgins et al., 2011), and non-randomized studies were assessed using the Risk of Bias in Non-randomized Studies of Interventions (ROBINS-I) (Sterne et al., 2016). There was 100% agreement between all raters on the overall risk of bias for each of the included studies. The certainty of primary and secondary outcomes included in the meta-analysis was assessed using the Cochrane Grading of Recommendations Assessment, Development, and Evaluation (GRADE) Framework (Guyatt et al., 2011).

## Results

### Study Characteristics

Of 1566 records identified, 36 full-text articles were screened, of which 11 were deemed eligible for inclusion. Nine studies provided sufficient data for meta-analysis (Asdigian et al., 2023; Beeres et al., 2022; Bonell et al., 2020; Brown et al., 2019; Haug et al., 2022; Kelder et al., 2020; Lisboa et al., 2019; Rozema et al., 2018; Weser et al., 2021). Table 1 provides study characteristics. Overall, the 11 articles included five cluster RCTs and six quasi-experimental trials. The studies comprised a total of 36,275 students, with sample sizes ranging from 158 to 13,269 students. Students were between 12 and 21 years at baseline, and 50.98% were female. Trials were conducted between 2014 and 2020, most commonly in the US (*n* = 5), and the length of follow-up varied from immediately post-test to 36 months. Comparison groups included assessment only (*n* = 6), education as usual (*n* = 4), and an education-only component of an intervention (*n* = 1). All studies used self-report survey measures to assess their outcomes. Baseline e-cigarette use was reported in six studies (Beeres et al., 2022; Bonell et al., 2020; Kelder et al., 2020; Rozema et al., 2018; Weser et al., 2021; Williams et al., 2022) with prevalence rates for lifetime use ranging from 0% (Williams et al., 2022) (due to only including never users at baseline) to 13.4% (Rozema et al., 2018). Three studies reported on past 30-day e-cigarette use at baseline, with prevalence rates of 2.2% (Lisboa et al., 2019), 4.3% (Okamoto et al., 2019), and 36% (Haug et al., 2022) (the latter being a combined measure of tobacco and e-cigarette use). Some studies (*n* = 6) also measured other substance use at baseline, which included tobacco use (*n* = 5), straw cigarettes (*n* = 1), water pipe/hookah (*n* = 4), alcohol (*n* = 2), marijuana (*n* = 2), and hard drugs and crystal meth (*n* = 1).

**Table 1:** Characteristics of included studies

Study characteristics				Sample characteristics	Measurement		Intervention				
Study	Design and setting	Comparison	Follow-up	*n*, mean age (SD), other	Primary Outcome of Systematic Review & Meta-Analysis	Secondary Outcomes of Systematic Review & Meta-Analysis	Program	Content and components	Frequency and duration	Theory	Other intervention components
Asdigian et al. 2022	Quasi-experimental (non-equivalent comparison group pilot study), 1 high school and 1 middle school, United States	No intervention	High school: immediately post-intervention Middle school: 1 month	High school: *n* = 51, *M*_age_ = N.R/48% aged 16, 54% female Middle school: *n* = 107, *M*_age_ (SD) not reported/48% aged 13, 63% female	N/A	Future intentions to vape (next 12 months), likelihood of vaping if offered, vaping susceptibility, resistance skills, knowledge	YES-CAN!	Classroom program that supports older adolescents (high schoolers) in developing and delivering short narrative prevention videos to younger adolescents (middle schoolers) The program the high school students received from their science teacher focused on public health messaging, video production, and presentations/discussion, along with material adapted from the Stanford Medicine Tobacco Prevention Toolkit on the history of the tobacco and vaping industries, the chemical composition and health risks of cigarette smoking and nicotine vaping, the health effects of nicotine, reasons youth vape, resistance skills, and stress management strategies High school students then presented their videos to 7th and 8th grade students and led discussions, answered questions, and shared additional information about vaping	(1) Science teacher delivered the program to high school students (10th to 12th grade)—10 weeks for 2.5 h per day (2) High school students presented their videos to 7th and 8th grade students during 1 × class	N.R, but draws on social influence principles	N/A
Beeres et al., 2022	Cluster randomized controlled trial, 34 schools, Sweden	Tobacco-Free Duo classroom education only	9 months, 21 months	*N* = 1176, *M*_age_ = N.R, age range 12–13 years, 51% female	E-cigarette ever-use (probability of remaining a never user)	Ever- and regular- use of tobacco, snus, and water pipe	Tobacco-Free Duo (T-Duo)	Multicomponent, school, and community-based program including a signed agreement between adolescents and adults (18 + years) to remain tobacco-free for 3 years [Tobacco-free pair (Duo) core component], student & parent information provided by school staff, school-specific membership cards for benefits for participating students, annual school organized prize draw for tobacco-free adolescents, and interactive classroom education conducted by trained school personnel in all classes during grades 6 (or 7) to 9	(1) Tobacco-free pair (Duo) 3-year contract between adolescent and adult (2) Student information (year 1, 1 × 30-min meeting) (3) Parent information (year 1, 1 × 30-min meeting) (4) Yearly membership card that entitles adolescents to fringe benefits (5) Yearly prize draw organized by school for adolescents affirming remained tobacco-free (6) Interactive classroom education (4 × per year, 1 h)	Social influence approach	N/A
Bonell et al., 2020	Cluster randomized controlled trial (2-arm parallel repeat cross-sectional), 40 schools, The UK	Education as usual (with a head teacher signed contract precluding their engagement with facilitated whole school programs)	24 months, 36 months	*N* = 6667, *M*_age_ = 12 years (0.4), 52.7% female	E-cigarette ever use (prevention/reduction of e-cigarette use)	Tobacco use, alcohol use, mental well-being, psychological functioning, and health-related quality of life	Learning Together/INCLUSIVE	Multicomponent, whole school intervention including: (1) Annual surveys of local needs and assets and progress in addressing these (2) Support from an external expert education facilitator trained in facilitating INCLUSIVE (3) Social and emotional learning curriculum resources (4) Staff training in restorative practices provided by the education facilitators and comprising a short introduction and subsequent half day for all staff (5) Convening of an action group that must meet at least six times per school year and develop an action plan that coordinates delivery of the intervention outputs: i. Reviewing and revising school rules and policies relating to discipline, behavior management, and staff-student communication ii. Implementing restorative practices throughout the school iii. Additional tailored actions to address local priorities iv. Delivering the social and emotional skills curriculum for years 8 to 10	5–10 h of lessons a year (students) 3 days training and action group meeting 2 × per term (teachers)	Theory of Human Functioning and School Organisation	N/A
Brown et al., 2019	Cluster randomized controlled trial, 2 middle schools, and 7 elementary schools, the United States	Education as usual	Pre- (1–19 days) and immediately post-intervention	*N* = 2257, *M*_age_ = N.R, 47.7% female	N/A	Tobacco susceptibility, outcome expectations (i.e., attitudes), knowledge, normative beliefs, and intentions	Teens Against Tobacco Use (TATU)	One-off presentation, delivered in-school by youth involved in the after-school program. The presentations focused on tobacco prevention and harms. High school youth presented to middle school physical education classes whereas middle school youth presented to 4th and 5th graders at nearby elementary schools	One-off 45-min presentation on a single day delivered in class	Social Cognitive Theory	This study examined the impact of one TATU presentation on 4th–8th grader tobacco susceptibility; however, the TATU intervention also includes youth involvement in tobacco control advocacy activities (e.g., policy and environmental change initiatives, attending/organizing tobacco prevention events, including a smoke-free parks cigarette clean-up event and the Texas Tobacco-Free Kids Day, and tobacco retailer compliance checks)
Haug et al., 2022	Cluster randomized controlled trial, 159 classes at vocational schools, Switzerland	No intervention	Baseline, 6 months	*N* = 1351, *M*_age_ = 17.3 years (3), 43.4% female	N/A	Past 30-day at-risk drinking, past 30-day tobacco/ e-cigarette use, past 30-day cannabis use, problematic internet use, number of alcoholic drinks consumed in past 30 days, number of tobacco cigarettes smoked in past 30 days, number of cannabis use days in past 30 days, general self-efficacy, self-perceived stress	Ready4life	Mobile app-based program that provides individualized chatbot coaching to promote life skills and reduce risk behaviors. Students pick two out of six possible coaching topics: i. Stress ii. Social skills iii. Social media and gaming iv. Tobacco/e-cigarette smoking, v. Cannabis vi. Alcohol Utilizing information from the baseline assessment, the virtual coach encouraged the participants to consume addictive substances responsibly, provided feedback on current use and life skills, and offered tailored information in weekly dialogs	Students receive app-based coaching for a period of 4-months (2 months per topic). An average weekly dialog took between 2 and 5 min to process	Social Cognitive Theory, Social Norms Approach, and Motivational Interviewing	“Ask the expert feature” on the app allowed students to ask questions of addiction prevention experts Interactive quizzes, challenges, and games were used to increase engagement Users could earn credits for completing a weekly dialog session and go in the draw to win a prize
Kelder et al., 2020	Quasi-experimental (Pretest–posttest, serial cross-sectional design), 12 schools, United States	Education as usual	Baseline, 4 months, 16 months	*N* = 2542, *M*_age_ = N.R, 45.2% female	E-cigarette ever-use	Past 30-day e-cigarette use, never users susceptibility to e-cigarette use, ever- and past 30-day tobacco use, e-cigarette knowledge, and attitudes	CATCH My Breath	6 activities taught via 4 classroom lessons overseen by the teachers who are trained via webinar to implement the program. All program activities are designed to require active participation led by trained peer facilitators who are elected by classmates Students: (1) Are educated (via group discussion) on the contents of e-cigarettes, the short-term health consequences of e-cigarette use, and school policies and age-of-sale restrictions (2) Compare their expectations of peers’ e-cigarette use with data on e-cigarette prevalence (normative education) (3) Explore reasons or motivations for e-cigarette use and discuss positive alternatives to achieve these goals (4) Learn how these reasons or motivations are established through social and environmental influences (5) Learn and practice refusal skills to resist social influences to use e-cigarettes and role-play possible social encounters (6) Make a public commitment to abstain from e-cigarette use	4 × 25 min classroom lessons	Social Cognitive Theory	Peer facilitators practice facilitating group activities with their teachers in a brief 30-min session before curriculum implementation. Classroom teachers ensure preparedness of each peer facilitator and routinely check in with peer facilitators to address any issues that may arise
Lisboa et al., 2019	Cluster randomized controlled trial (group allocation at the class level), 14 schools, Brazil	No intervention	Baseline, 6 months, 12 months	*N* = 2348, *M*_age_ = 14.8 years (SD N.R), 52.7% female	N/A	Smoking prevalence (difference in the change between intervention and control groups), prevention of smoking onset, quitting smoking	Education Against Tobacco (EAT), including using the “Smokerface” app	The 90-min classroom program is delivered by 2 medical students and involved discussing features of smoking that students can relate to in a gain-framed and interactive manner, provision of age-appropriate information to help students reframe a positive nonsmoking image, and use of a three-dimensional facial-aging app, “Smokerface” developed by EAT Students form 4 groups and rotate to 4 different stations in the classroom: i. Discusses different tobacco products and extraction of substances of tobacco smoke ii. Attractiveness and photoaging consequences of nonsmoking and mechanisms related to the face are discussed iii. Performance benefits of nonsmoking iv. Student’s own experiences with tobacco and how they reacted in the past to peer pressure and to the strategies of the tobacco industry to influence their decision	One-off 90-min module presented in class	Theory of Planned Behaviour	N/A
Okamoto et al., 2019	Quasi-experimental (dynamic wait-listed control group design), 13 schools, the United States	No intervention	Cohort 1: 3 months, 9 months, 12 months, 15 months Cohort 2: 2 months, 6 months, 9 months, 12 months Cohort 3: 3 months	*N* = 486, *M*_age_ = N.R, 52.1% female	N/A	Tobacco/e-cigarette use, alcohol use, marijuana use, crystal methamphetamine use, hard drug use	Ho’ouna Pono Drug Prevention Curriculum	Classroom-based, video-enhanced curriculum delivered by teachers, with lessons primarily focused on resistance skills training The curriculum is centered on brief video vignettes of Hawaiian youth engaged in realistic drug-related problem situations and these are matched to a set of three different resistance strategies. Core drug resistance strategies covered in the curriculum include i. Overt refusal of drug offers ii. Explaining the reasons behind drug refusal iii. Avoiding situations where drugs might be present iv. Redirecting the topic away from drug use v. Leaving a situation where drugs are present Eight of the nine lessons incorporate one 4–7-min video vignette. All lessons in the curriculum followed the same basic format: i. An introduction and/or review of the past lesson ii. A culture wall activity iii. A video iv. 1–2 interactive activities v. A wrap-up activity	9 × 45–60-min classroom lessons	N.R, but draws on social influence principles	N/A
Rozema et al., 2018	Quasi-experimental, 19 schools, The Netherlands	No intervention	Baseline, 6 months, 18 months	*N* = 5742, *M*_age_ = 13.7 years (1.1), 47.4% female	E-cigarette ever use (with and without nicotine)	Tobacco cigarette smoking prevalence and onset, water pipe ever use	Unnamed outdoor smoking bans	School policy implemented to ban smoking outdoors on school grounds	N/A—school-level policy	N.R	N/A
Weser et al., 2021	Quasi-experimental (non-equivalent control groups design), 12 classroom units, the United States	Education as usual	1 week, 3 months, 6 months	*N* = 279, *M*_age_ = 12.5 years (0.6), 46.6% female	E-cigarette ever use	E-cigarette knowledge, nicotine addiction knowledge, perceptions of e-cigarette use, perceived likelihood of e-cigarette use, harm perceptions, refusal self-efficacy, social approval, social perceptions	Invite Only VR: A Vaping Prevention Game	Virtual reality, story-based video game delivered in class to teach students about the health risks of using e-cigarettes and provide the opportunity to practice refusal skills. The game covers peer pressure, refusal skills, and correcting misperceptions	Students play Invite Only VR for 2–4 sessions for approximately 40 min per session in regular physical education class (those who opted to take fewer breaks completed it in fewer sessions)	Theory of Planned Behaviour & Social Cognitive Theory	N/A
Williams et al., 2021	Quasi-experimental (longitudinal pre-post study), 88 schools, Canada	No intervention	12 months	*N* = 13,269, *M*_age_ = N.R, 56% female	E-cigarette ever use (initiation at follow-up)	N/A	N/A—evaluating a range of school-based e-cigarette and tobacco prevention and cessation programs/initiatives delivered across different schools	Schools implemented various programs and initiatives across 4 categories: (1) Cessation • A vaping focus group and a vaping information and cessation program were developed and offered • Tobacco Enforcement Officers to speak with small groups of students to discuss cessation. Additionally, the school nurse set up displays from a variety of agencies to assist in smoking cessation • Activities related to “I stop, I win” and a policy for a tobacco-free generation (CQLT). Additionally, awareness activities were done in the classroom (2) Cessation; prevention • A new tobacco prevention program, cessation program, and vaping prevention program but no details were provided (NEI) • NEI is a new tobacco prevention program, cessation program, and vaping prevention program • Theme week—hired a new school nurse to help with cessation. The school nurse also implemented a tobacco-free week (3) Prevention • Interactive display—external organization presented interactive stations to illustrate the results of e-cigarette/vaping use • Interactive display—had Health Canada present their vaping workshop and display • Interactive display—had Health Canada do a class presentation and activity • Interactive display—put up posters and had students participate in a vaping maze • NEI—worked with their public health nurse and unit to deliver programming to students and parents • NEI—psycho-educator implemented prevention programs against vaping • NEI—increased promotion and vigilance around anti-vaping messages • NEI—a new vaping prevention program • NEI—a new vaping prevention program • NEI—a new vaping prevention program • Theme week—an Addiction Prevention Week • Presentation—a group of teachers who led classroom sessions using videos, guest speakers, and Tobacco Enforcement Officers to discuss the harmful effects of vaping • Presentation—invited police officers to come in and talk about substance abuse and related issues • Presentation—various sessions including spotlights, community education, parent information nights, and round table discussions • Presentation—workshops with the help of a special education technician • Presentation—prevention workers discuss the laws around vaping (4) Protection • Mandatory online awareness program and quiz about vaping and a suspension re-entry program for vaping • Community School Resource Officer and a Tobacco Enforcement Officer spoke to students and staff about fines for vaping and smoking on school property	Varied across schools	N.R	N/A

*SD* standard deviation, *N/A* not applicable, *N.R* not reported

### Intervention Characteristics

Most interventions targeted e-cigarette and tobacco use concurrently (*n* = 8) (Asdigian et al., 2023; Beeres et al., 2022; Brown et al., 2019; Haug et al., 2022; Kelder et al., 2020; Lisboa et al., 2019; Okamoto et al., 2019; Rozema et al., 2018); however, one (Weser et al., 2021) targeted e-cigarette use alone, and another (Bonell et al., 2020) focused more broadly on transforming the school environment through restorative approaches to address conflict, behavior management policies, and social and emotional learning. Additionally, in one study (Williams et al., 2022), multiple prevention and cessation programs and policy interventions were evaluated across 24 schools. Although the descriptions were limited, the interventions appeared to vary substantially (e.g., some focused on e-cigarettes and others included tobacco more broadly; some involved teacher-led sessions and others involved external facilitators, police officers, or school nurses; some involved classroom education and others involved presentations, eHealth components, school policy, or theme weeks). Among the other eligible studies, interventions included in-class student information and skills training programs led by peers and/or teachers (*n* = 6) (Asdigian et al., 2023; Brown et al., 2019; Kelder et al., 2020; Lisboa et al., 2019; Okamoto et al., 2019; Weser et al., 2021), multicomponent programs (*n* = 2) (Beeres et al., 2022; Bonell et al., 2020), school-wide policy (*n* = 1) (Rozema et al., 2018), and a mobile phone app (*n* = 1) (Haug et al., 2022). Delivery methods comprised face-to-face (*n* = 2) (Bonell et al., 2020; Brown et al., 2019), eHealth-only (*n* = 2) (Haug et al., 2022; Weser et al., 2021), hybrid (*n* = 5) (Asdigian et al., 2023; Beeres et al., 2022; Kelder et al., 2020; Lisboa et al., 2019; Okamoto et al., 2019), and policy-only (*n* = 1) (Rozema et al., 2018) formats. All studies evaluated universal preventive interventions; however, the one study that evaluated multiple programs across schools (Williams et al., 2022) also included cessation programs for students already using e-cigarettes or tobacco cigarettes. Intervention duration and frequency ranged from a one-off 45-min presentation (Brown et al., 2019) to a 3-year tobacco-free contract between adolescents and an adult (Beeres et al., 2022). Most interventions (*n* = 9) were underpinned by behavioral theory, spanning: social cognitive theory (*n* = 4); a social influence approach (*n* = 3), the theory of planned behavior (*n* = 2); the theory of human functioning and school organization (*n* = 1); the social norms approach (*n* = 1); and/or motivational interviewing (*n* = 1).

### Risk of Bias and Quality of Evidence

Risk of bias assessments is provided in Supplemental Figs. S1 and S2. Overall, we judged the quality of evidence to be very low to moderate (see summary table in Supplemental Table S2). Among randomized studies, one (Haug et al., 2022) was judged at high risk of bias for past 30-day tobacco use due to missing outcome data, and there were some concerns for the remaining studies. This was primarily due to a lack of reporting of methods and missing outcome data. Among non-randomized studies, one (Asdigian et al., 2023) was judged at serious risk of bias across outcomes due to bias in the classification of interventions and outcome measurement. There was also a moderate risk of bias judgement for all other studies, primarily due to potential bias in the measurement of outcomes, again related to insufficient availability of pre-specified protocols, and confounding.

### Outcomes

#### Aim 1—Evaluate the Efficacy of School-Based Preventive Interventions in Preventing E-Cigarette Use and Improving Secondary Outcomes

##### Primary Outcome: The Prevention of E-Cigarette Use at Longest Follow-Up

**Fig. 2:**
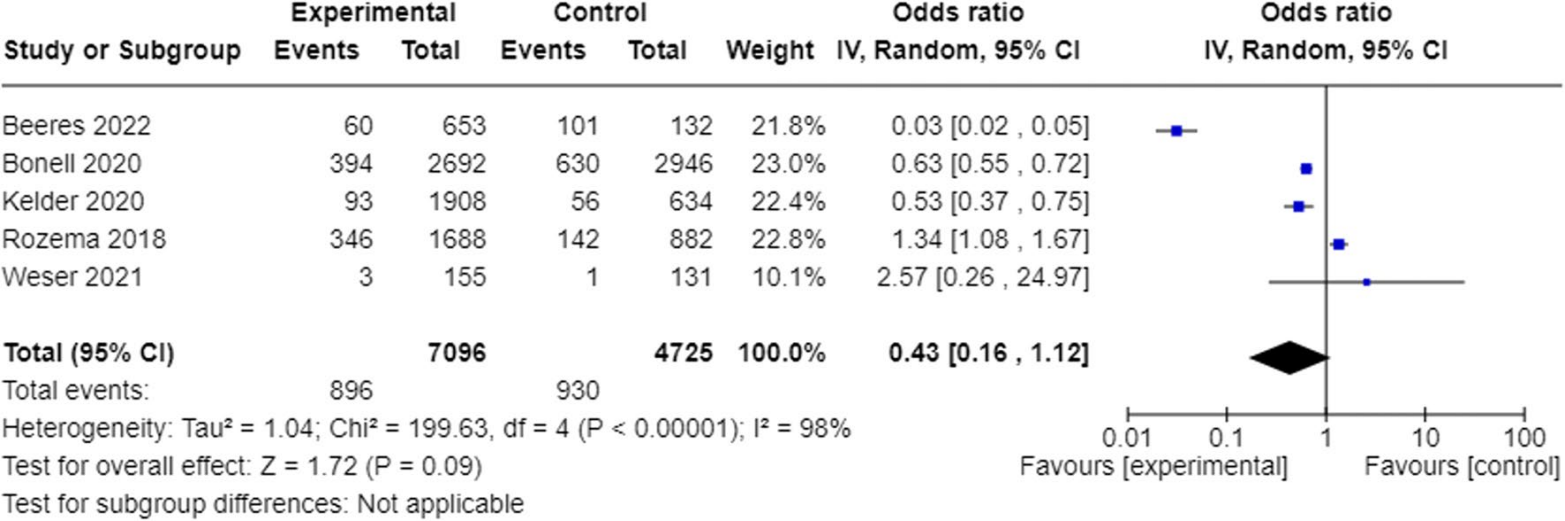
The prevention of e-cigarette use at the longest follow-up (ranging from 6 to 36 months)

Of the six studies reporting specifically on the prevention of e-cigarette use (Beeres et al., 2022; Bonell et al., 2020; Kelder et al., 2020; Rozema et al., 2018; Weser et al., 2021; Williams et al., 2022), five included primary outcome data sufficient for meta-analysis (see Fig. 2). Overall, we did not find evidence that school-based interventions were associated with the prevention of e-cigarette use at the longest follow-up (ranging between 6 and 36 months post-intervention: OR = 0.43, 95% CI = 0.16, 1.12; *p* = 0.09; GRADE rating: very low). There was considerable heterogeneity between studies (*I*^2^ = 98%, *p* < 0.00001, Tau^2^ = 1.04), with the prediction interval of the overall effect ranging from OR = 0.06 to 3.31. The quality of evidence was downgraded to very low due to substantial heterogeneity between studies and imprecision. Subgroup and sensitivity analyses indicated that results varied based on follow-up timepoint: the overall effect became significant when limited to post-test assessments only (OR = 0.40, 95% CI 0.17 to 0.96, *I*^2^ = 98%, *p* < 0.00001, Tau^2^ = 0.85, prediction interval of overall effect: OR = 0.06 to 2.53; Fig. S3) and when studies with follow-up < 12 months were omitted (OR = 0.35, 95% CI 0.13 to 0.97, *I*^2^ = 98%, *p* < 0.00001, Tau^2^ = 1.04; prediction interval of overall effect: OR = 0.05 to 2.69; Fig. S4); however, substantial heterogeneity and uncertainty in the underlying effects remained. Further subgroup analysis found no difference in the overall effect estimate or degree of heterogeneity when examined based on intervention type (education and skills training alone versus broader school initiatives; Fig. S5) or study design (RCT vs quasi-experimental; Fig. S6). All studies were rated as having a moderate risk of bias.

#### Secondary Outcomes

##### Tobacco use

Seven trials examined changes in tobacco use—the definition of which varied across studies, e.g., referring to tobacco cigarettes specifically (Beeres et al., 2022; Bonell et al., 2020; Rozema et al., 2018) or encompassing e-cigarettes and/or other types such as cigars, pipes, and hookah in other studies (Haug et al., 2022; Kelder et al., 2020; Lisboa et al., 2019; Okamoto et al., 2019)–of which five studies provided data sufficient for meta-analysis (Beeres et al., 2022; Haug et al., 2022; Kelder et al., 2020; Lisboa et al., 2019; Rozema et al., 2018). Pooled analysis showed that overall, school-based interventions were associated with a significant reduction in past 30-day tobacco use at the longest follow-up (three studies; OR = 0.59, 95% CI = 0.39, 0.89, *p* = 0.01; GRADE rating: low, see Fig. S7). However, there was substantial and significant heterogeneity between trials (*I*^2^ = 79%, *p* = 0.009, Tau^2^ = 0.10) and the prediction interval of the overall effect ranged from OR = 0.31 to 1.11. The quality of evidence was downgraded to low due to risk of bias and inconsistency between studies. Subgroup and sensitivity analyses revealed the overall effect did not differ when examined at post-test only (Fig. S8), or by study design (RCT vs quasi-experimental; Fig. S9), however was no longer significant when the one study with follow-up < 12 months and high risk of bias was omitted (Haug et al., 2022; Fig. S10). Intervention and control groups did not significantly differ in terms of lifetime tobacco use (two studies, OR = 1.01, 95% CI = 0.65, 1.59, *p* = 0.95, GRADE rating: moderate), with considerable inconsistency between the two studies (*I*^2^ = 86%, *p* = 0.008 Tau^2^ = 0.09, prediction interval: 0.55 to 1.84; Fig. S11). Results did not differ when examined at post-test only (Fig. S12). No further subgroup or sensitivity analyses were feasible as there were only two studies in this analysis.

##### Knowledge

School-based interventions had a small, yet significant effect on improving knowledge about e-cigarettes compared to controls at post-test (four studies, SMD =  − 0.38, 95% CI =  − 0.68, − 0.08, *p* = 0.01; GRADE rating: moderate, Fig. S13). However, there was considerable heterogeneity between studies (*I*^2^ = 78%, *p* = 0.004, Tau^2^ = 0.06, prediction interval: SMD =  − 0.87 to 0.11). Sensitivity and subgroup analyses revealed no change in the overall effect when examining outcomes at the longest follow-up only (Fig. S14) or based on study design (RCT vs quasi-experimental; Fig. S15); however, the effect was no longer significant when studies at high risk of bias were omitted (Fig. S16).

##### Intentions to use e-cigarettes/tobacco

Overall, school-based interventions were associated with a significant reduction in students’ intentions to use e-cigarettes/tobacco at post-test (four studies, SMD =  − 0.15, 95% CI =  − 0.22, − 0.07, *p* = 0.0001, GRADE rating: moderate), with no heterogeneity between studies (*I*^2^ = 0%; *p* = 0.40, Tau^2^ = 0; Fig. S17). Sensitivity and subgroup analyses revealed no impact of follow-up timepoint (longest assessment; Fig. S18), study design (RCT vs quasi-experimental, Fig. S19), or risk of bias (high risk omitted, Fig. S20) on the outcome.

##### Risky attitudes

Pooled analysis found a small, significant effect of school-based interventions on reducing risky attitudes towards e-cigarette use at post-test (three studies, SMD =  − 0.14, 95% CI =  − 0.22, − 0.06; *p* = 0.0007; GRADE rating: moderate), with little variability between the three studies (*I*^2^ = 0%, *p* = 0.39, Tau^2^ = 0; Fig. S21). Subgroup analyses were not feasible as all studies reported post-test data only and had the same design.

##### Harm perceptions

Overall, there was no clear evidence that school-based preventive interventions changed the harm perceptions of adolescents at post-test (SMD =  − 0.29, 95% CI =  − 0.73, 0.15, *p* = 0.20; GRADE rating: moderate, Fig. S22). There was substantial and significant heterogeneity between trials (*I*^2^ = 69%, *p* = 0.04, Tau^2^ = 0.10). Sensitivity analyses revealed that this effect did become significant at the longest follow-up (SMD =  − 0.44, 95% CI − 0.66 to − 0.22, *I*^2^ = 0%, Tau^2^ = 0.00; Fig. S23). No other subgroup analyses were able to be conducted.

#### Aim 2—Identify and Summarize the Key Components and Characteristics Associated with Efficacious Interventions

##### Primary Outcome: The Prevention of E-Cigarette Use at Longest Follow-Up

Overall, three of the six studies reporting specifically on the prevention of e-cigarette use found significant intervention effects in the expected direction and were categorized as efficacious interventions for the purposes of narrative synthesis (Beeres et al., 2022; Bonell et al., 2020; Kelder et al., 2020).

##### Program type

Of these three studies, two used multicomponent programs (Beeres et al., 2022; Bonell et al., 2020), consisting of school-based components (e.g., classroom education and teacher training) plus broader initiatives involving parents or external adults. The other (Kelder et al., 2020) involved classroom education and skills training alone, with teacher- and peer-led components.

##### Intervention duration

In all three studies, classroom education was delivered over multiple sessions, ranging between 4 × 25 min lessons and a 10-h learning curriculum.

##### Delivery method

Two interventions were delivered via hybrid format (face-to-face and online; Beeres et al., 2022; Kelder et al., 2020), and one was face-to-face only (Bonell et al., 2020).

##### Portion dedicated to e-cigarette prevention

Two interventions (Beeres et al., 2022; Kelder et al., 2020) focused specifically on e-cigarettes/tobacco, whereas the other (Bonell et al., 2020) focused on broader behavior management via social and emotional learning.

##### Theoretical underpinning

Two interventions were based on social cognitive theory (*n* = 2) (Beeres et al., 2022; Kelder et al., 2020) and the other on the theory of human functioning and school organization (*n* = 1) (Bonell et al., 2020).

Outside of these three studies, it is important to note that iatrogenic effects were reported in one study (Williams et al., 2022), where participants reported higher odds of e-cigarette initiation following the implementation of a broad “theme week.” While there was limited information on the intervention components, it did not appear to include specific classroom education/skills training or be grounded in any specific theory.

#### Secondary Outcomes

##### Tobacco Use

Of the seven trials that examined change in tobacco use, five reported significant intervention effects in the expected direction and were included in the narrative synthesis (Beeres et al., 2022; Bonell et al., 2020; Kelder et al., 2020; Lisboa et al., 2019; Okamoto et al., 2019). Three of these were the same studies that demonstrated significant effects on the prevention of e-cigarette use (Beeres et al., 2022; Bonell et al., 2020; Kelder et al., 2020).

##### Program type

Three were school-only educational and skills programs (Kelder et al., 2020; Lisboa et al., 2019; Okamoto et al., 2019) and two used multicomponent programs (Beeres et al., 2022; Bonell et al., 2020) which incorporated parents, whole of school initiatives, and the broader community (e.g., information nights).

##### Intervention duration

Only one intervention was a standalone “one-off” program of 90 min duration (Lisboa et al., 2019), with the remainder being multi-session programs with the number of sessions ranging from 4 to 10 (Kelder et al., 2020; Okamoto et al., 2019).

##### Delivery method

Four interventions were delivered in a hybrid format, comprising both face-to-face and online/eHealth or video components (Beeres et al., 2022; Kelder et al., 2020; Lisboa et al., 2019; Okamoto et al., 2019) and one was face-to-face only (Bonell et al., 2020). In terms of intervention facilitators, two of the five incorporated peers alongside teachers (Kelder et al., 2020; Lisboa et al., 2019), and the remainder were led by program-trained teachers (Beeres et al., 2022; Bonell et al., 2020; Okamoto et al., 2019), with Bonell et al. (2020) additionally incorporating external facilitators.

##### Theoretical underpinnings

There was no one consistent theoretical underpinning of these studies, with theories including the social cognitive theory (*n* = 2) (Beeres et al., 2022; Kelder et al., 2020), the theory of planned behavior (*n* = 1) (Lisboa et al., 2019), the theory of human functioning and school organization (*n* = 1) (Bonell et al., 2020), and, although not explicitly stated, social influence principles (*n* = 1) (Okamoto et al., 2019).

##### Portion dedicated to tobacco prevention

In three studies, tobacco use was broadly defined and encompassed cigarettes, e-cigarettes, and/or other types of tobacco such as cigars, pipes, and hookah (Kelder et al., 2020; Lisboa et al., 2019; Okamoto et al., 2019). The remaining two studies referred to tobacco cigarettes specifically (Beeres et al., 2022; Bonell et al., 2020). Importantly, iatrogenic effects were reported in one of these studies (Rozema et al., 2018), with participants reporting greater tobacco smoking onset following the implementation of a school-wide anti-smoking policy. Similar to the other study reporting iatrogenic effects on e-cigarette onset (Williams et al., 2022), this intervention did not appear to include classroom education/skills training or to be grounded in any specific theory.

##### Knowledge, Attitudes, Normative Beliefs, and Harm Perceptions

Four studies were associated with a significant improvement in theoretical constructs related to e-cigarette and/or tobacco cigarette use, including knowledge (Asdigian et al., 2023; Brown et al., 2019; Kelder et al., 2020; Weser et al., 2021), attitudes (Asdigian et al., 2023; Brown et al., 2019; Kelder et al., 2020), normative beliefs (Asdigian et al., 2023), harm perceptions (Weser et al., 2021), and future intentions to use tobacco (Brown et al., 2019). All of these interventions centered on classroom education and skills training; however, some involved teacher- and peer-led components delivered over multiple lessons via a hybrid format (Asdigian et al., 2023; Kelder et al., 2020), one involved a one-off peer-led face-to-face presentation (Brown et al., 2019), and the other comprised an eHealth program used over multiple lessons (Weser et al., 2021). The most common theoretical underpinning was the social cognitive theory (*n* = 3) (Brown et al., 2019; Kelder et al., 2020; Weser et al., 2021), with two interventions also drawing on social influence principles (*n* = 1) (Asdigian et al., 2023) and the theory of planned behavior (*n* = 1) (Weser et al., 2021).

##### Mental Health

In terms of other secondary outcomes, two studies were associated with significantly better mental health outcomes, including lower perceived stress (Haug et al., 2022) and greater mental wellbeing, psychological functioning, and health-related quality of life (Bonell et al., 2020). Although both studies incorporated student education and skills training over multiple sessions, one did so via face-to-face format within a broader multicomponent intervention that additionally comprised staff training and school policies and practices (Bonell et al., 2020), while the other used an eHealth intervention delivered outside of class (Haug et al., 2022). Both interventions were also associated with reduced alcohol use.

##### Substance Use

Finally, two interventions were associated with the prevention or reduction of illicit drug use, including the broader multicomponent intervention focusing on social and emotional learning (Bonell et al., 2020) and another intervention similarly involving student education and skills training over multiple sessions (Okamoto et al., 2019). There were no clear characteristics of successful studies in terms of theoretical underpinnings.

## Discussion

This is the first systematic review and meta-analysis to examine the efficacy of school-based preventive interventions for e-cigarette use. Our meta-analysis found no evidence that school-based interventions were significantly associated with the prevention of e-cigarette use at the longest follow-up, which ranged between 6 and 36 months post-intervention. However, effects were significant when examined at post-test and again when studies with follow-up < 12 months were omitted. Our narrative synthesis supported this mixed finding and showed that three (Beeres et al., 2022; Bonell et al., 2020; Kelder et al., 2020) of the six trials found significant intervention effects in the expected direction, while one intervention inadvertently increased e-cigarette use (Williams et al., 2022). When considering the prevention of tobacco use more broadly, which encompassed e-cigarettes in some studies, our meta-analysis showed that school-based interventions were associated with a significant reduction in past 30-day tobacco use at the longest follow-up, albeit with substantial and significant heterogeneity between studies. No effect was found for lifetime tobacco use. Our narrative synthesis found that five trials (Beeres et al., 2022; Bonell et al., 2020; Kelder et al., 2020; Lisboa et al., 2019; Okamoto et al., 2019) reported significant intervention effects on tobacco use in the expected direction, whereas one increased tobacco smoking onset (Rozema et al., 2018). Our meta-analyses found that school-based interventions were significantly associated with improvements in knowledge, intentions, and attitudes towards e-cigarettes and tobacco at post-test. Our narrative synthesis supported these findings and also revealed favorable effects on mental health outcomes (Bonell et al., 2020; Haug et al., 2022) and other substance use (Bonell et al., 2020; Haug et al., 2022; Okamoto et al., 2019). We judged the certainty in the body of evidence to be very low-to-moderate overall.

Overall, our findings suggest that e-cigarette prevention programs may have a transient beneficial effect on preventing e-cigarette use; however, there was too much underlying variability in the study designs, interventions, and follow-up assessment points to have strong certainty in this finding. This highlights the need for more high-quality research to develop effective prevention programs for e-cigarette use and to measure outcomes over the long-term, as has been demonstrated for alcohol and other drug use (Newton et al., 2022; Rowland et al., 2019; Thomas et al., 2013). The findings of the narrative synthesis, though mixed, suggest that classroom education and skills training delivered over multiple sessions (ranging between 4 × 25 min lessons and 10 h learning curriculum) were consistently associated with reductions in e-cigarette use onset. This finding broadly aligns with the established recommendation that school-based programs for tobacco, alcohol, and other drugs should include developmentally appropriate information along with resistance and social skills training over a series of structured sessions (Botvin & Griffin, 2007). Two interventions (Beeres et al., 2022; Bonell et al., 2020) also included broader components, such as parental/adult involvement. For example, the core component of the “Tobacco-Free Duo” intervention (Beeres et al., 2022) is a formal agreement between the student and a parent/adult to be tobacco-free for 3 years. A previous review of parent-based interventions found mixed results for the prevention of adolescent tobacco and alcohol use, with some interventions producing iatrogenic effects (Champion et al., 2022). However, the parent intervention components were more intensive than those in the present review and were typically not delivered as part of a broader school-based intervention.

The three interventions identified through the narrative synthesis as being efficacious in preventing e-cigarette use (Beeres et al., 2022; Bonell et al., 2020; Kelder et al., 2020) simultaneously produced improvements related to tobacco use, as did two additional interventions which included e-cigarettes within their broad definition of tobacco (Lisboa et al., 2019; Okamoto et al., 2019). When tobacco outcomes were meta-analyzed, school-based interventions were only associated with improvements in past 30-day tobacco use, but there was no evidence for a significant association with lifetime use. This likely relates to the small number of studies eligible for inclusion in the meta-analysis. Moreover, given previous reviews have demonstrated that school-based interventions can prevent tobacco use (Thomas et al., 2015), this may also suggest that current interventions targeting both e-cigarettes and tobacco need to be improved upon. This could be done by ensuring alignment with the principles of effective school-based substance use prevention (Botvin & Griffin, 2007) or by improving engagement and implementation fidelity, which few studies reported on.

Notably, two interventions were associated with iatrogenic effects on the prevention of e-cigarette use (Williams et al., 2022) and tobacco smoking onset (Rozema et al., 2018). This included a “theme week” (Williams et al., 2022) and a school-wide policy which also produced null effects on e-cigarette use (Rozema et al., 2018). From the limited descriptions, both interventions appeared broad, without specific classroom education and skills training, nor a theoretical underpinning. It is possible that these interventions raised awareness of, but did not impart sufficient skills to prevent the onset of e-cigarette and tobacco use, which aligns with prior evidence that education alone is not sufficient to prevent substance use (Stockings et al., 2016). These findings highlight the critical importance of ensuring schools implement evidence-based programs to prevent e-cigarette/tobacco use.

Despite the mixed evidence generated for the prevention of e-cigarette and tobacco use, the meta-analyses indicated that school-based interventions were associated with significant improvements in knowledge, intentions, and attitudes. These constructs are theorized precursors of behavior change (Ajzen, 1991; Bandura, 1986), yet, in the two studies that examined these constructs along with actual e-cigarette or tobacco use, only one (Kelder et al., 2020) observed positive intervention effects on all outcomes, which was at 16-month post-intervention timepoint. The other intervention, “Invite Only VR” (Weser et al., 2021), was associated with improved knowledge, but did not have a significant effect on e-cigarette use. This may have been due to the follow-up period only extending to 6 months, as a previous review found the effects of tobacco preventive interventions tend to only emerge after 1 year (Thomas et al., 2015). Nevertheless, the four interventions identified via the narrative synthesis as having positive effects across knowledge (Asdigian et al., 2023; Brown et al., 2019; Kelder et al., 2020; Weser et al., 2021), attitudes (Asdigian et al., 2023; Brown et al., 2019; Kelder et al., 2020), normative beliefs (Asdigian et al., 2023), harm perceptions (Weser et al., 2021), and future intentions to use tobacco (Brown et al., 2019), all involved classroom education and skills training that was grounded in theory, again highlighting the potential of this intervention strategy. Three of the interventions (Asdigian et al., 2023; Brown et al., 2019; Kelder et al., 2020) also involved peer-led components, which has previously been shown to be effective at addressing adolescent substance use when compared to adult-led education (Mellanby et al., 2000). The use of eHealth intervention components, either in hybrid (Asdigian et al., 2023; Kelder et al., 2020) or eHealth-only (Weser et al., 2021) format, with delivery over multiple sessions also appeared beneficial.

Finally, although there were insufficient data for meta-analyses, the narrative synthesis found some interventions were associated with improvements in mental health (Bonell et al., 2020; Haug et al., 2022) and other substance use, including reduced alcohol (Bonell et al., 2020; Haug et al., 2022) and illicit drug use (Bonell et al., 2020; Okamoto et al., 2019). This is promising, given the strong associations between mental health and adolescent substance use, including e-cigarette use (Truong & Cotton, 2023). Indeed, two of these studies (Bonell et al., 2020; Okamoto et al., 2019) reported simultaneous improvements in e-cigarette and/or tobacco use, both of which involved classroom education and skills training over multiple sessions.

### Limitations and Future Directions

Despite the rigorous methodology, this study had several limitations, including the small number of studies and significant heterogeneity present in many of our meta-analyses. The high heterogeneity limits the ability to make meaningful comparison of key intervention characteristics and their efficacy. However, it was important to examine these characteristics given it is common for school-based preventive interventions to vary substantially in type, duration, and frequency in real-world educational settings (Champion et al., 2019, 2022). The quality of evidence was also very low or low for some outcomes, and effects were generally small and short-lived. This underscores the need for future high-quality RCTs, with prospective registration of trial protocols required to reduce potential bias. Only 6 out of the 11 identified studies measured actual e-cigarette and/or tobacco cigarette use, and there was substantial variation in the definitions across studies, making it difficult to tease out effects specifically on e-cigarettes compared to tobacco cigarettes and other tobacco products. Timeframes for the longest follow-up were also highly variable, ranging between 6 (Weser et al., 2021) and 36 months (Bonell et al., 2020). Longer-term follow-up periods are important when evaluating substance use preventive interventions, as effects are most likely to emerge in later adolescence as exposure to substances increases (Guerin & White, 2018; Thomas et al., 2015). The remainder of the studies focused on theoretical constructs related to e-cigarette/tobacco use; however, to fully understand the efficacy of such interventions in preventing e-cigarette use, an assessment of actual substance use is required.

## Conclusions

Although some studies demonstrated that school-based e-cigarette preventive interventions can produce positive effects, some interventions negatively impacted students, highlighting the critical importance of supporting schools to identify and adopt evidence-based programs. Overall, our meta-analyses on a small subsample of studies found no evidence that school-based interventions prevented e-cigarette or tobacco use at the longest follow-up, which ranged between 6 and 36 months post-intervention. However, some evidence of effect was identified at immediate post-test, and when only follow-ups > 12 months were considered. We also found significant reductions in past 30-day tobacco use, which encompassed e-cigarettes in some studies. School-based interventions were also associated with improvements in knowledge, intentions, and attitudes, with some individual studies reporting improvements in mental health outcomes and other substance use. Classroom education and student skills training that is grounded in theory and delivered by trained teachers were commonly associated with beneficial program effects. Peer-led and eHealth components also appeared promising to improve precursors of behavior change; however, more research, over the longer-term, is required to evaluate whether this translates into effects on e-cigarette use. Given previous research has demonstrated that school-based interventions can be effective in preventing tobacco, alcohol, and other drug use, school-based interventions may have unmet potential for addressing e-cigarette use. More high-quality research is required to develop efficacious interventions, and schools must be supported to adopt evidence-based programs.

## Supplementary Information

Below is the link to the electronic supplementary material.

Supplementary file1 (DOCX 1461 kb)

## Data Availability

Data are available upon reasonable request to the study team.

## Footnotes

1 New legislation came into effect 1 July 2024. Prior to this, any person wishing to legally purchase a nicotine-containing vape was required to hold a prescription from their General Practitioner, and nicotine containing e-cigarettes could then be purchased either via community pharmacy or online by the Therapeutic Goods Administration Personal Importation Scheme, which as of 1 July 2024 is defunct.
