# Supplementary material for: A Systematic Review and Meta-analysis of School-Based Preventive Interventions Targeting E-Cigarette Use Among Adolescents
Source: Prev Sci. 2024 Sep 26;25(7):1104–21. doi: 10.1007/s11121-024-01730-6 (PMC11519311; doi:10.1007/s11121-024-01730-6)
Supplement: Supplementary file 1 — Supplementary file1 (DOCX 1461 kb) [file 11121_2024_1730_MOESM1_ESM.docx]

**Supplemental Material**

Table of Contents

[Table S1: Example search strategy (CINAHL) 3](#_Toc174104026)

[Table S2: Summary of findings table (quality of evidence ratings) 4](#_Toc174104027)

[Figure S1- Risk of Bias of Randomised Studies 6](#_Toc174104028)

[Figure S2- Risk of Bias of Non-Randomised Studies 7](#_Toc174104029)

[Additional figures for subgroup and sensitivity analyses 8](#_Toc174104030)

[**Figure S3** - Sensitivity analysis for the prevention of e-cigarette use: Post-test timepoint only 8](#_Toc174104031)

[**Figure S4-** Sensitivity analysis for the prevention of e-cigarette use: Omitting studies with follow-up <12-months (remaining studies ranged from 12- to 36-months) 8](#_Toc174104032)

[**Figure S5-** Subgroup analysis for the prevention of e-cigarette use at longest follow up (6- to 36-months): Intervention type (student education/skills training alone versus broader interventions) 9](#_Toc174104033)

[**Figure S6 -** Subgroup analysis for the prevention of e-cigarette use at longest follow up (6- to 36-months): Study design (RCT vs quasi-experimental) 9](#_Toc174104034)

[**Figure S7-** Overall effect for past 30-Day tobacco use at longest follow-up 10](#_Toc174104035)

[**Figure S8** - Sensitivity analysis for past 30-Day tobacco use: Post-test timepoint only 10](#_Toc174104036)

[**Figure S9 -** Subgroup analysis for past 30-Day tobacco use: Study design (RCT vs quasi-experimental) 11](#_Toc174104037)

[**Figure S10 -** Subgroup analysis for past 30-Day tobacco use: Omitting studies with <12-month follow-up/high risk of bias 11](#_Toc174104038)

[**Figure S11**- Overall effect for lifetime tobacco use at longest follow-up 12](#_Toc174104039)

[**Figure S12** - Sensitivity analysis for lifetime tobacco use: Post-test timepoint only 12](#_Toc174104040)

[**Figure S13** – Overall effect for knowledge about e-cigarettes at the first post-test timepoint 12](#_Toc174104041)

[**Figure S14** - Sensitivity analysis for knowledge about e-cigarettes: Longest follow-up timepoint only 12](#_Toc174104042)

[**Figure S15**- Subgroup analysis for knowledge about e-cigarettes: Study design (RCT vs. quasi-experimental) 13](#_Toc174104043)

[**Figure S16**- Sensitivity analysis for knowledge about e-cigarettes: Omitting studies with high risk of bias 13](#_Toc174104044)

[**Figure S17**- Overall effect for intentions to use e-cigarettes at the first post-test timepoint 13](#_Toc174104045)

[**Figure S18-** Sensitivity analysis for intentions to use e-cigarettes: Longest-follow-up timepoint only 14](#_Toc174104046)

[**Figure S19-** Subgroup analysis for intentions to use e-cigarettes: Study design (RCT vs quasi-experimental) 14](#_Toc174104047)

[**Figure S20-** Sensitivity analysis for intentions to use e-cigarettes: Omitting studies at high risk of bias 14](#_Toc174104048)

[**Figure S21**- Overall effect for risky attitudes towards e-cigarettes at the first post-test timepoint 15](#_Toc174104049)

[**Figure S22**- Overall effect for harm perceptions at the first post-test timepoint 15](#_Toc174104050)

[**Figure S23**- Sensitivity analysis for harm perceptions: Longest-follow-up timepoint only 15](#_Toc174104051)

# Table S1: Example search strategy (CINAHL)

| S1 | (MH “Vaping”) | 688 |
| --- | --- | --- |
| S2 | TI ( vaping OR vape OR vapes OR "electronic nicotine delivery system*" OR ecig* OR "e-cig*" OR (electr* N2 cig*) OR juul OR juuls OR "nicotine vapo?r*" OR "heated tobacco product*" OR eliquid* OR "e-liquid" OR enicotine OR "e-nicotine" OR ejuice OR "e-juice" OR "cigarette liquid" OR "electronic vapo?r*" OR ehookah* OR "e-hookah*" OR eshisha* OR "e-shisha*" OR ewaterpipe* OR "e-waterpipe*" OR "electr* hookah*" ) OR AB ( vaping OR vape OR vapes OR "electronic nicotine delivery system*" OR ecig* OR "e-cig*" OR (electr* N2 cig*) OR juul OR juuls OR "nicotine vapo?r*" OR "heated tobacco product*" OR eliquid* OR "e-liquid" OR enicotine OR "e-nicotine" OR ejuice OR "e-juice" OR "cigarette liquid" OR "electronic vapo?r*" OR ehookah* OR "e-hookah*" OR eshisha* OR "e-shisha*" OR ewaterpipe* OR "e-waterpipe*" OR "electr* hookah*" ) | 7,103 |
| S3 | S1 OR S2 | 7,196 |
| S4 | (MH "Young Adult") OR (MH "Adolescence+") OR (MH "Child") OR (MH "Minors (Legal)") | 1,011,191 |
| S5 | TI ( child OR children OR teen* OR adolescent* OR youth* OR juvenile* OR minor OR preadult* OR "pre adult*" OR (young N2 (adult* OR person* OR people* OR individual* OR man OR men OR wom?n)) OR (emerging N2 (adult* OR person* OR people)) ) OR AB ( child OR children OR teen* OR adolescent* OR youth* OR juvenile* OR minor OR preadult* OR "pre adult*" OR (young N2 (adult* OR person* OR people* OR individual* OR man OR men OR wom?n)) OR (emerging N2 (adult* OR person* OR people)) ) | 765,302 |
| S6 | S4 OR S5 | 1,317,773 |
| S7 | (MH "Schools") OR (MH "Schools, Elementary") OR (MH "Schools, Middle") OR (MH "Schools, Secondary") OR (MH "Students, High School") OR (MH "Students, Middle School") OR (MH "Students, Elementary") OR (MH "Students") | 61,940 |
| S8 | TI ( school* N2 (middle OR high OR secondary OR based) ) OR AB ( school* N2 (middle OR high OR secondary OR based) ) | 44,693 |
| S9 | S7 OR S8 | 88,025 |
| S10 | S3 AND S6 AND S9 | 661 |

# Table S2: Summary of findings table (quality of evidence ratings)

| **Certainty assessment** | | | | | | | **№ of patients** | | **Effect** | | **Certainty** | **Importance** |
| --- | --- | --- | --- | --- | --- | --- | --- | --- | --- | --- | --- | --- |
| **№ of studies** | **Study design** | **Risk of bias** | **Inconsistency** | **Indirectness** | **Imprecision** | **Other considerations** | **[intervention]** | **[comparison]** | **Relative (95% CI)** | **Absolute (95% CI)** |  |  |
| Lifetime e-cigarette use at longest f/u | | | | | | | | | | | | |
| 5 | randomised trials | not serious | very serious^a^ | not serious | serious^b^ | none | 896/7096 (12.6%) | 930/4725 (19.7%) | **OR 0.43** (0.16 to 1.12) | **101 fewer per 1,000** (from 159 fewer to 19 more) | ⨁◯◯◯ Very low |  |
| Past 30 day tobacco use at longest f/u | | | | | | | | | | | | |
| 3 | randomised trials | serious | serious^a^ | not serious | not serious | none | 320/3340 (9.6%) | 363/1906 (19.0%) | **OR 0.59** (0.39 to 0.89) | **69 fewer per 1,000** (from 106 fewer to 17 fewer) | ⨁⨁◯◯ Low |  |
| Lifetime tobacco use at longest f/u | | | | | | | | | | | | |
| 2 | randomised trials | not serious | serious^a^ | not serious | not serious | none | 1626/3275 (49.6%) | 874/2142 (40.8%) | **OR 1.01** (0.65 to 1.59) | **2 more per 1,000** (from 99 fewer to 115 more) | ⨁⨁⨁◯ Moderate |  |
| Knowledge at post-test | | | | | | | | | | | | |
| 4 | randomised trials | serious | serious^a^ | not serious | not serious | none | 1313 | 1342 | - | SMD **0.38 SD lower** (0.68 lower to 0.08 lower) | ⨁⨁◯◯ Low |  |
| Outcome expectations/attitudes at post-test | | | | | | | | | | | | |
| 3 | randomised trials | serious | not serious | not serious | not serious | none | 1171 | 1216 | - | SMD **0.14 SD lower** (0.22 lower to 0.06 lower) | ⨁⨁⨁◯ Moderate |  |
| Intentions at post-test | | | | | | | | | | | | |
| 4 | randomised trials | serious | not serious | not serious | not serious | none | 1313 | 1342 | - | SMD **0.15 SD lower** (0.22 lower to 0.07 lower) | ⨁⨁⨁◯ Moderate |  |
| Harm perceptions at post-test | | | | | | | | | | | | |
| 3 | randomised trials | serious | serious^a^ | not serious | not serious | none | 212 | 186 | - | SMD **0.29 SD lower** (0.73 lower to 0.15 higher) | ⨁⨁◯◯ Low |  |

**CI:** confidence interval; **OR:** odds ratio; **SMD:** standardised mean difference

Explanations

a. High and significant heterogeneity

b. Wide confidence intervals, small sample size

# Figure S1- Risk of Bias of Randomised Studies


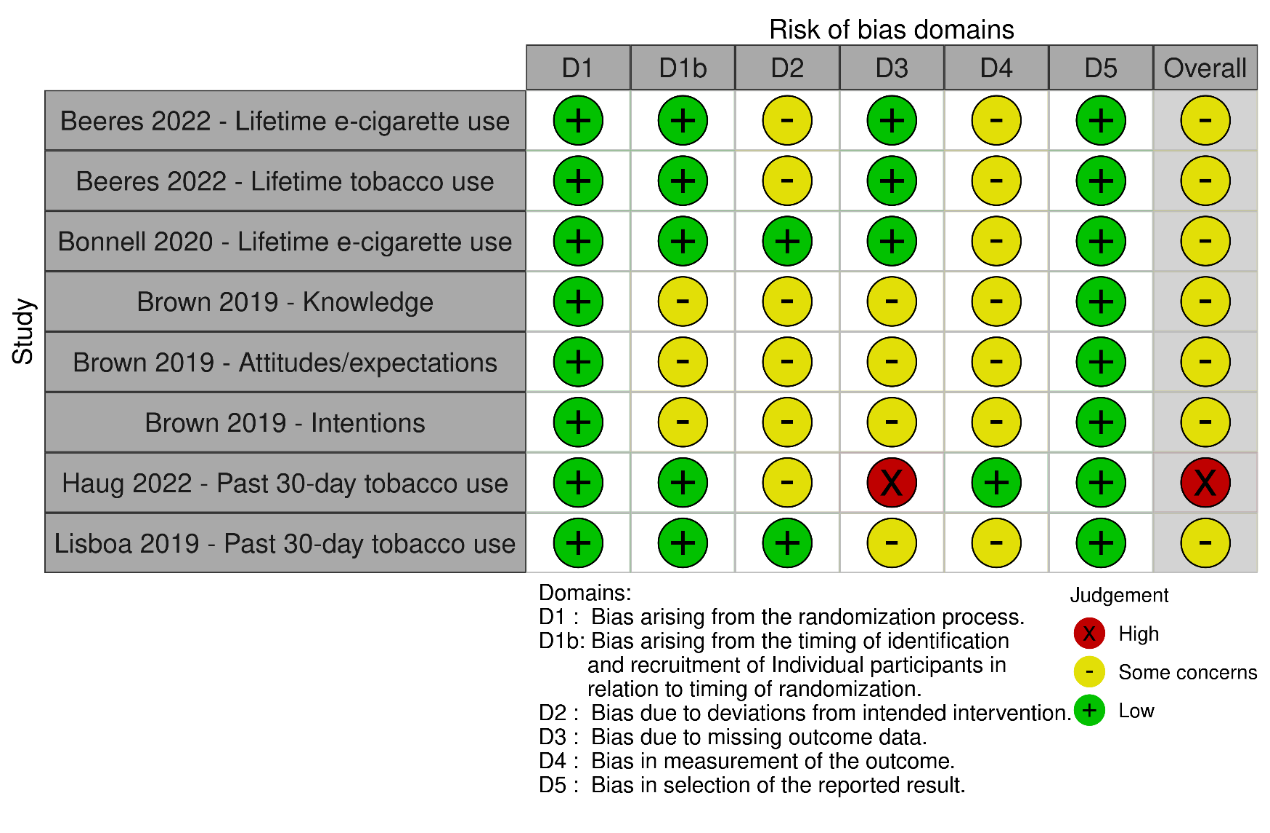


# **Figure S2- Risk of Bias of Non-Randomised Studies**


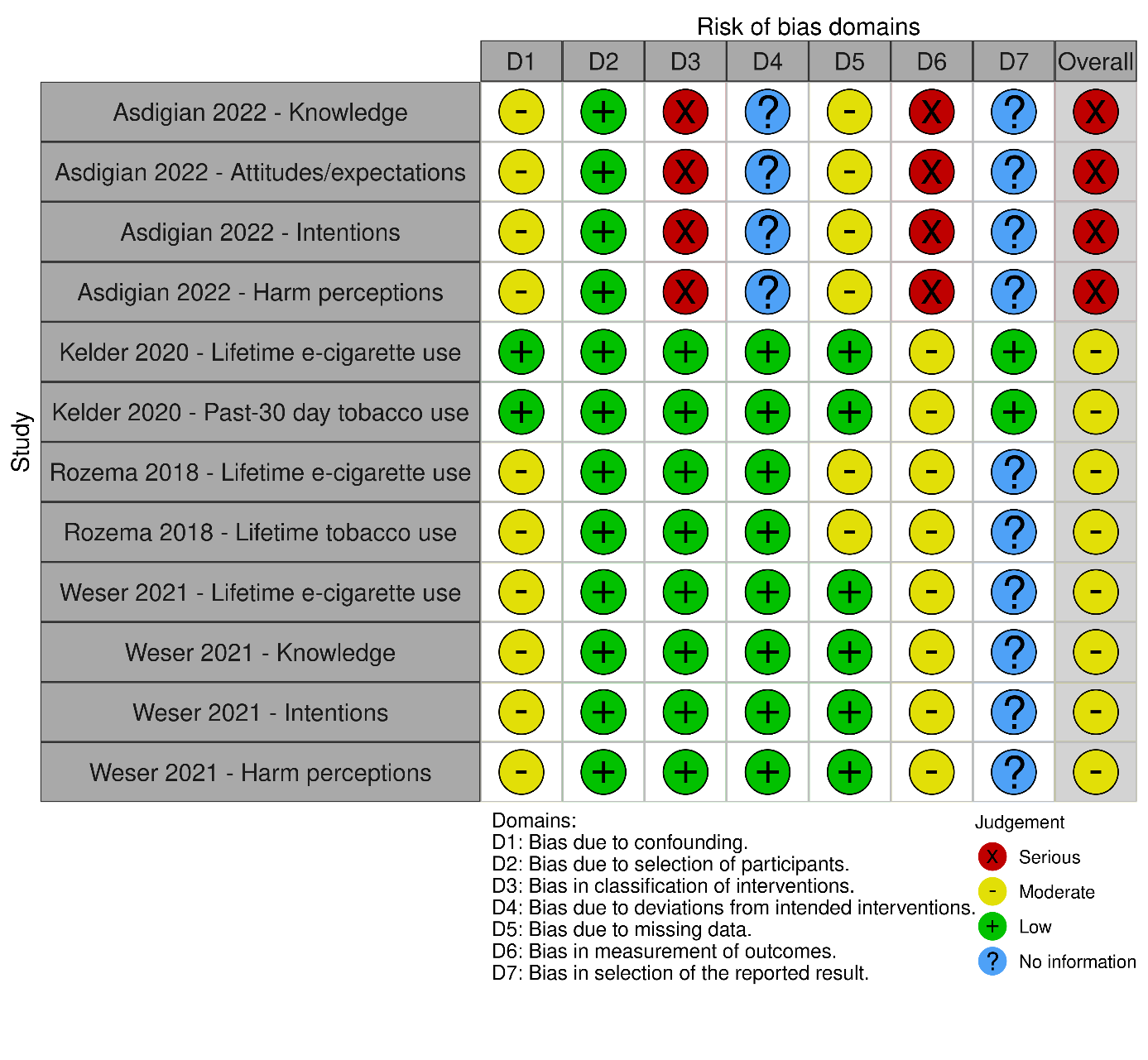


# Additional figures for subgroup and sensitivity analyses

## **Figure S3** - Sensitivity analysis for the prevention of e-cigarette use: Post-test timepoint only


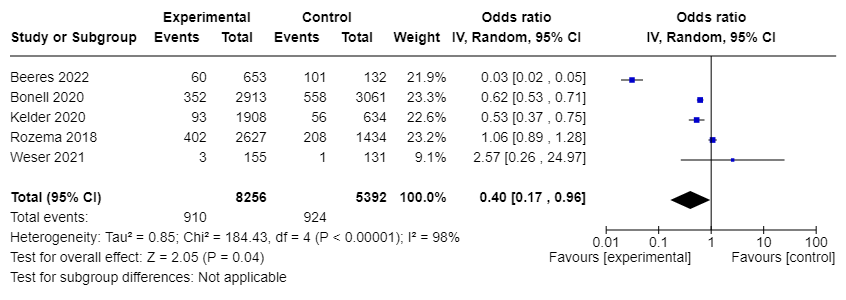


## **Figure S4-** Sensitivity analysis for the prevention of e-cigarette use: Omitting studies with follow-up <12-months (remaining studies ranged from 12- to 36-months)

*
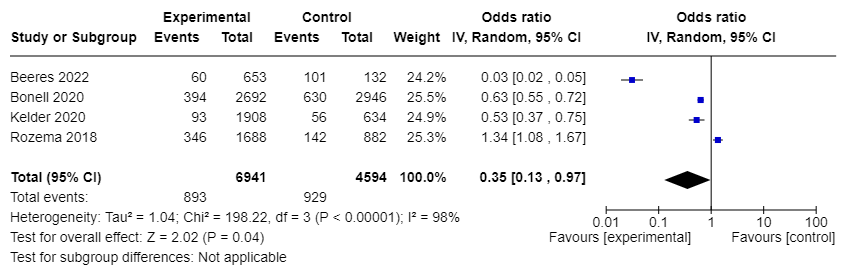
*

## **Figure S5-** Subgroup analysis for the prevention of e-cigarette use at longest follow up (6- to 36-months): Intervention type (student education/skills training alone versus broader interventions)


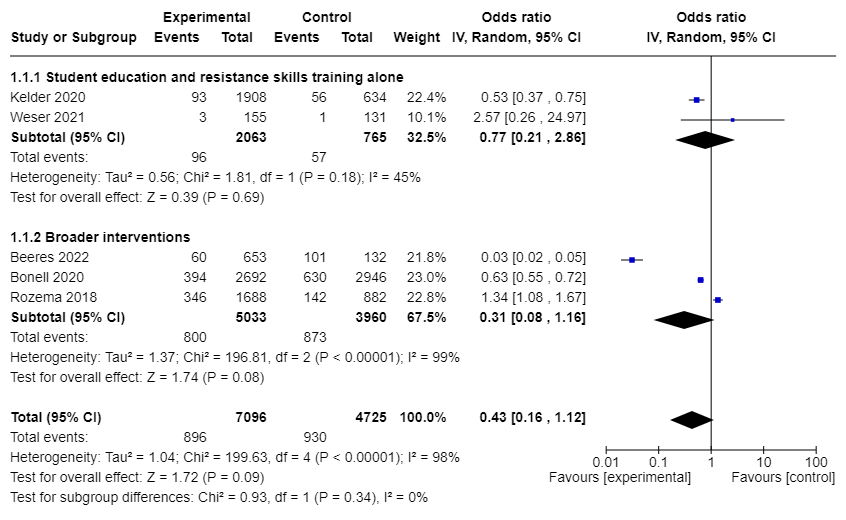


## **Figure S6 -** Subgroup analysis for the prevention of e-cigarette use at longest follow up (6- to 36-months): Study design (RCT vs quasi-experimental)

*
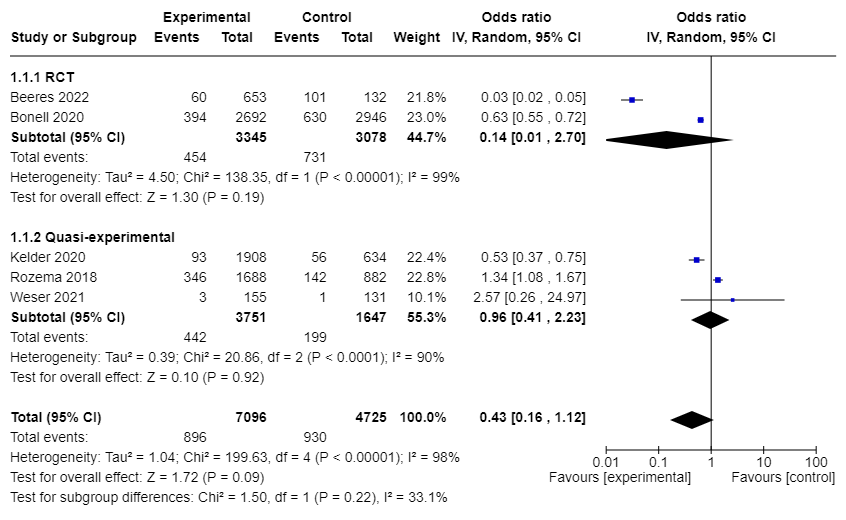
*

## **Figure S7-** Overall effect for past 30-Day tobacco use at longest follow-up


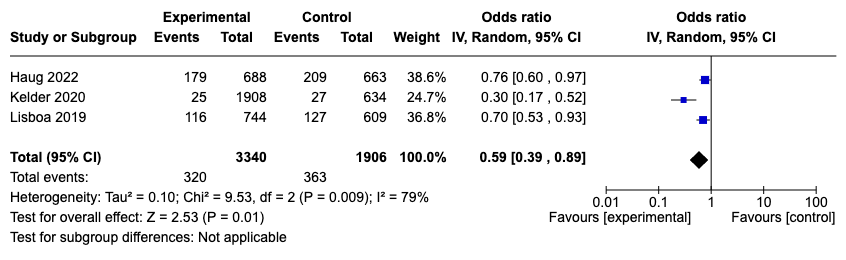


## **Figure S8** - Sensitivity analysis for past 30-Day tobacco use: Post-test timepoint only

**
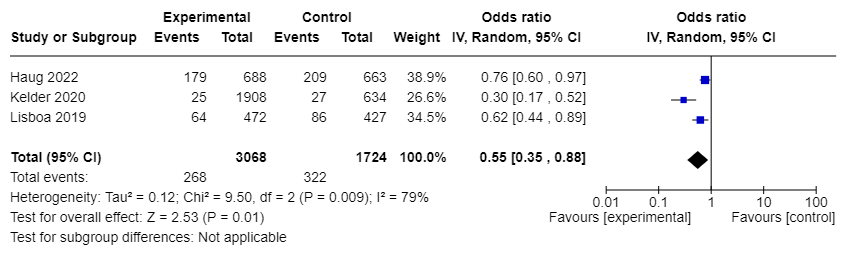
**

## **Figure S9 -** Subgroup analysis for past 30-Day tobacco use: Study design (RCT vs quasi-experimental)

**
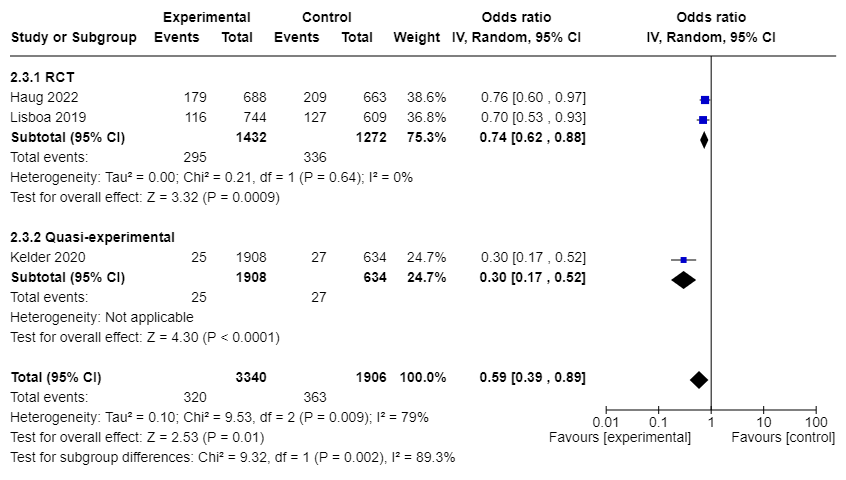
**

## **Figure S10 -** Subgroup analysis for past 30-Day tobacco use: Omitting studies with <12-month follow-up/high risk of bias

**
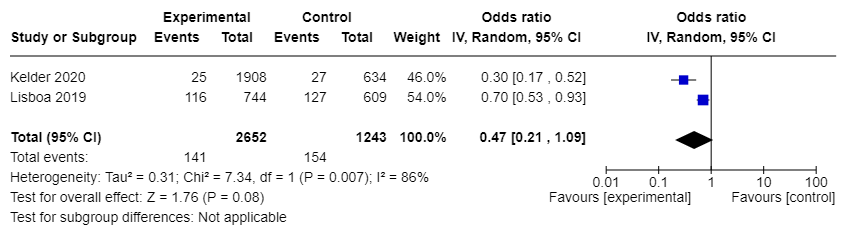
**

Note: This graph presents the effects of omitting one study (Haug 2022), as it met both criteria of collecting data at < 12 months post-intervention and having an overall rating of high risk of bias.

## **Figure S11**- Overall effect for lifetime tobacco use at longest follow-up


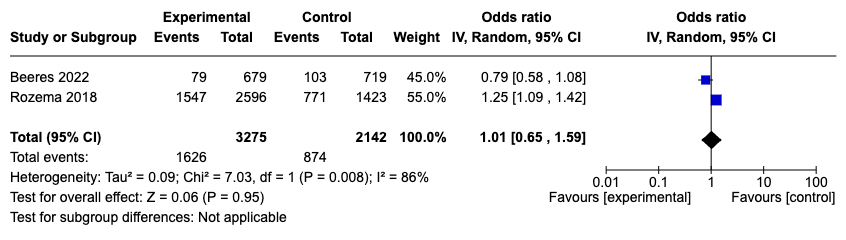


## **Figure S12** - Sensitivity analysis for lifetime tobacco use: Post-test timepoint only

**
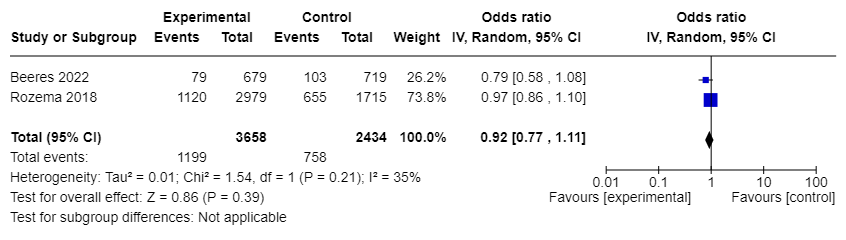
**

## **Figure S13** – Overall effect for knowledge about e-cigarettes at the first post-test timepoint


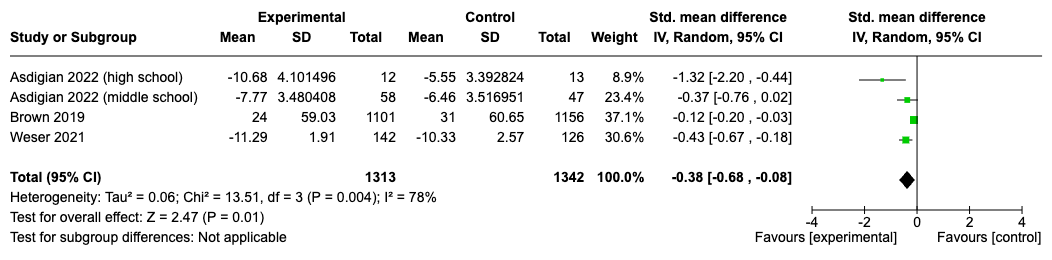


## **Figure S14** - Sensitivity analysis for knowledge about e-cigarettes: Longest follow-up timepoint only


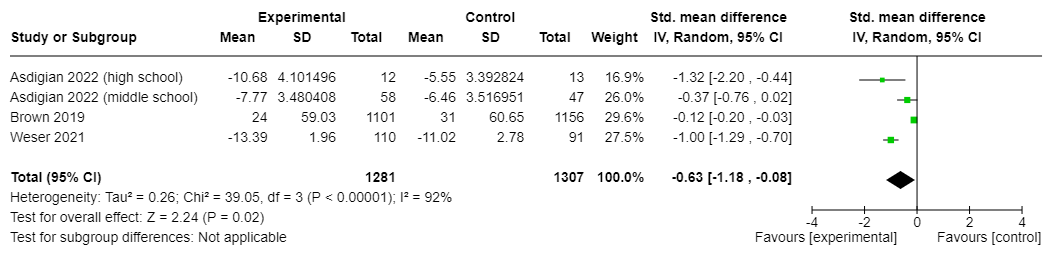


## **Figure S15**- Subgroup analysis for knowledge about e-cigarettes: Study design (RCT vs. quasi-experimental)


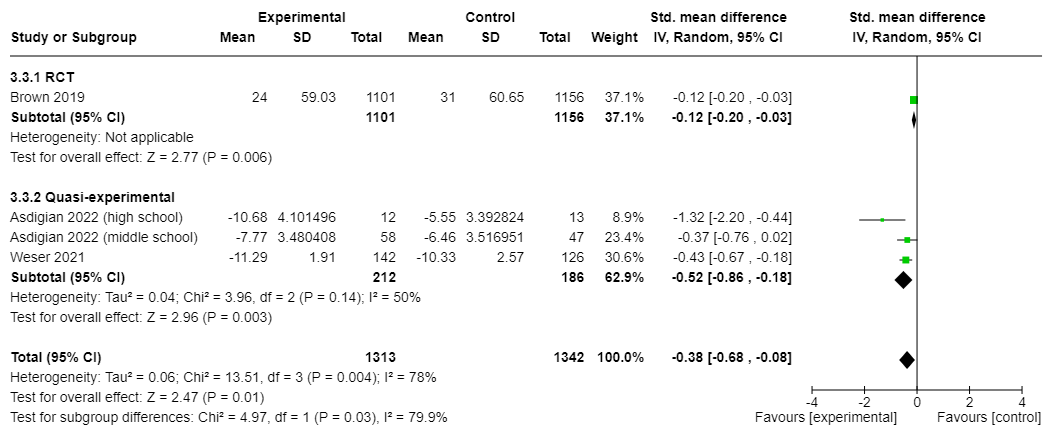


## **Figure S16**- Sensitivity analysis for knowledge about e-cigarettes: Omitting studies with high risk of bias


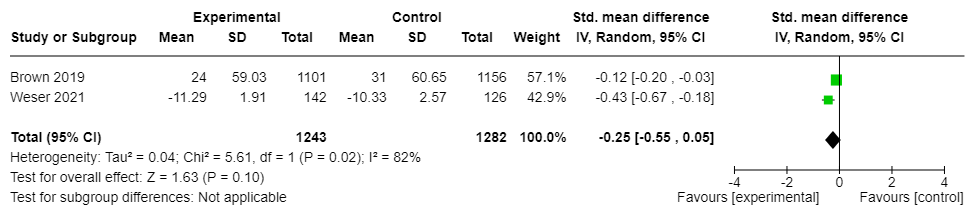


## **Figure S17**- Overall effect for intentions to use e-cigarettes at the first post-test timepoint


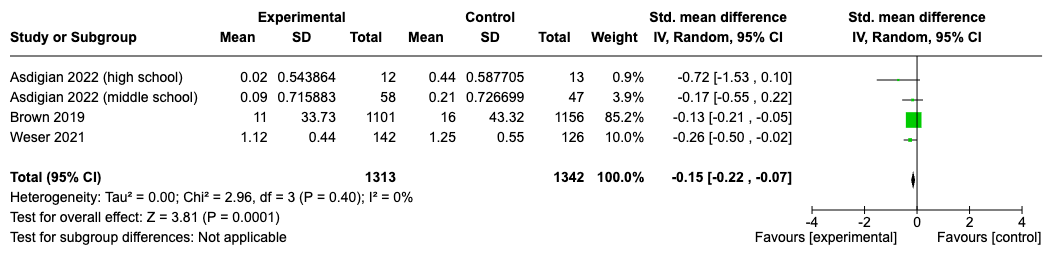


## **Figure S18-** Sensitivity analysis for intentions to use e-cigarettes: Longest-follow-up timepoint only

**
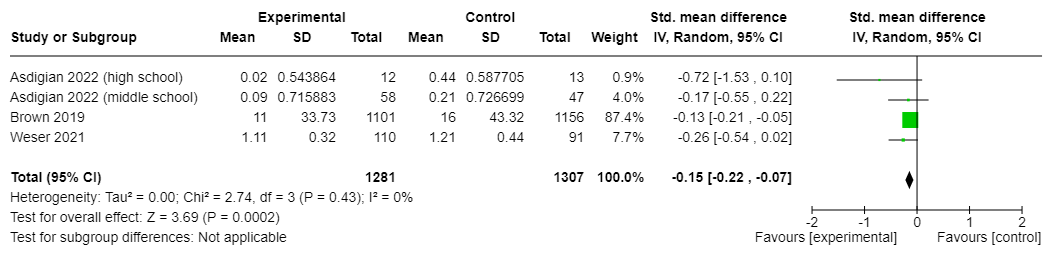
**

## **Figure S19-** Subgroup analysis for intentions to use e-cigarettes: Study design (RCT vs quasi-experimental)

**
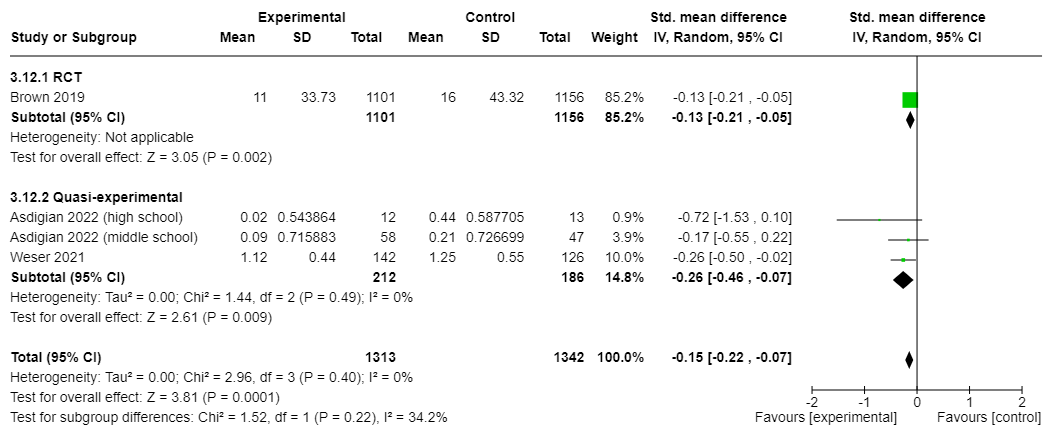
**

## **Figure S20-** Sensitivity analysis for intentions to use e-cigarettes: Omitting studies at high risk of bias

**
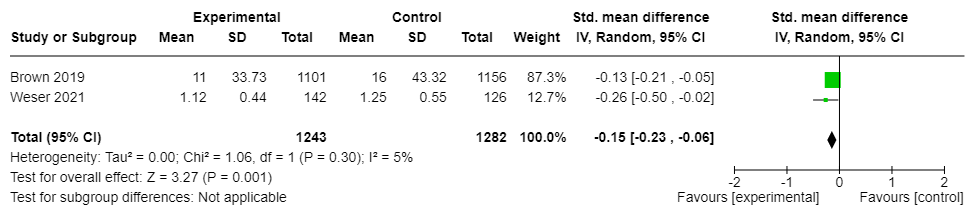
**

## **Figure S21**- Overall effect for risky attitudes towards e-cigarettes at the first post-test timepoint


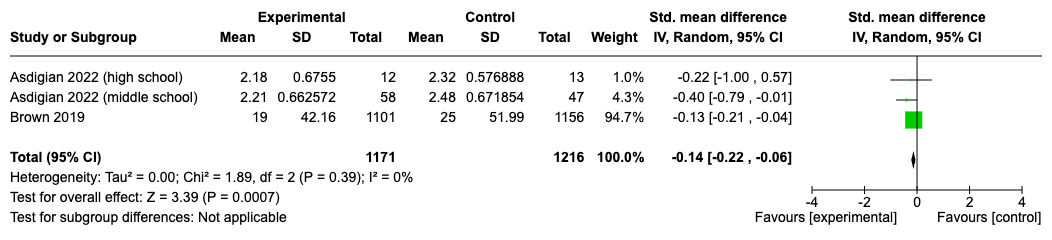


## **Figure S22**- Overall effect for harm perceptions at the first post-test timepoint


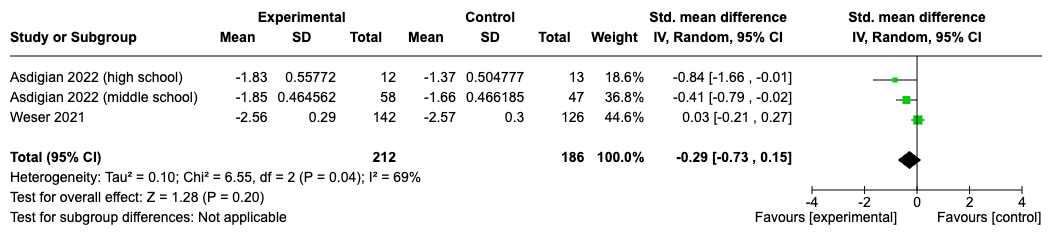


## **Figure S23**- Sensitivity analysis for harm perceptions: Longest-follow-up timepoint only

**
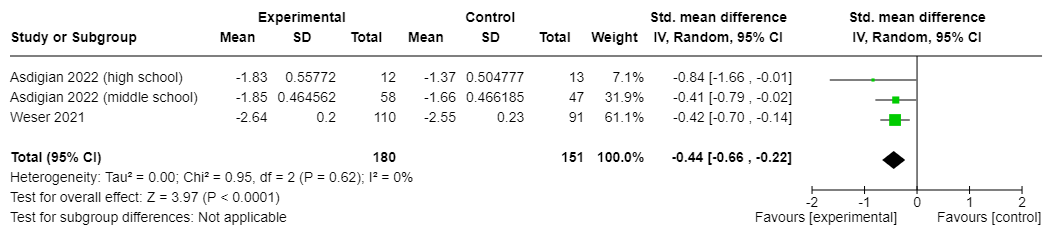
**
